# Femoral head fractures systematic review and meta-analysis

**DOI:** 10.1007/s00590-026-04821-y

**Published:** 2026-06-13

**Authors:** Samuel C. Marshall, Jinpu Li, Emily V. Leary, Caleb J. Bischoff, Ashwin R. Garlapaty, Brett D. Crist

**Affiliations:** 1 Department of Orthopaedic Surgery, University of Oklahoma, Norman, United States; 2 Department of Orthopaedic Surgery, University of Missouri, Columbia, United States; 3 Department of Orthopaedic Surgery, University of Missouri, Columbia, United States

**Keywords:** Femoral head fractures, Systematic review, Meta-analysis, Adverse outcomes

## Abstract

**Objective:**

Femoral head fractures are rare, high-energy injuries with a relatively high complication rate. This study evaluated the femoral head fracture literature published since the systematic review in 2009 and determined changes in: classification systems, management of these injuries, and the associated adverse outcomes.

**Methods:**

A PubMed search from January 2009 to January 2025 was performed. Terms associated with femoral head fractures were entered. Meta-analysis was performed for the pooled proportion of patients who experienced adverse outcomes in the evaluated studies. Heterogeneity between studies was tested using the Q test and, if significant, random effect models were applied. Confidence intervals from individual studies and stabilized pooled proportions are reported.

**Results:**

Twenty-two articles met inclusion criteria. All the studies supplied the mechanism of injury as well as patient age and sex. The most common mechanism of injury was traffic accidents in all the 22 studies with many of the injured middle-aged men. The Pipkin classification was used in 86.4% of studies. Open reduction and internal fixation (ORIF) and fragment excision were the two most common surgical techniques used. Adverse outcomes for patients included heterotopic ossification, post-traumatic osteoarthritis, and avascular necrosis with rates from 9 to 37%, across studies. For the rate of adverse outcomes, heterogeneity between studies was identified using a random effects model.

**Conclusion:**

Since 2009, the Pipkin classification system remains the most often used for femoral head fractures and ORIF is the most common treatment method. Although femoral head fractures are rare, the adverse outcome rate following injury is extremely high (35%). However, no relationships between fracture type, treatment type, and favorable outcome were identified.

## Introduction

Femoral head fractures, first described by Birkett [1] in 1869, are a rare entity complicated by inconsistencies in classification criteria, surgical approach, and treatment, including time to treatment and technique [2–10]. Femoral head fracture are often associated with a hip dislocation—75% posteriorly and 18% anteriorly [11]. Due to small incidence rates of femoral head fractures, evidence-based medical care typically relies on either multi-center, retrospective cohort studies, or systematic reviews with meta-analysis of case series or retrospective cohort studies.

Femoral head fracture classification is somewhat controversial, as none of the multiple classification systems which describe these fractures demonstrate superior utility [3, 4, 7, 12]. In 1954, Stewart and Milford classified fracture dislocations into four “grades”, in reference to the severity of the fracture, and two “types” in reference to the position of the dislocation [4]. First described in 1957, Pipkin’s classification divided the previously used Stewart and Milford’s Grade IV fracture dislocations into four additional grades specific for femoral head fracture dislocations [4, 5]. Subsequently, Pipkin’s classification scheme has been commonly used [2, 3, 5, 6, 8, 10, 13–19]. However, despite classifying posterior hip dislocations with femoral head fractures, the Pipkin classification fails to account for anterior or central dislocations, as well as the variation of acetabular fractures [3, 5, 8]. Chiron’s classification system addresses this by dividing femoral head fractures into five grades according to head fragment size [5, 12, 20, 21] and isolated fractures, associated acetabular fractures, or associated femoral neck fractures [12, 21]. Pascarella et al. used their own classification system that describes different fracture patterns in relation to different types of hip dislocation. There are four main groups of dislocation that Pascarella uses: Anterior, central, posterior, and bilateral dislocations. These are further divided into subgroups based on fracture pattern [15]. Brumback’s classification scheme includes posterior, anterior, and central dislocations as well as acetabular fractures and associated injuries, but it is infrequently used [3, 5, 8, 20]. Brumback’s system addresses the absence of fracture patterns and prognosis of anterior and central hip dislocations, as well as the unique differentiation of acetabular fractures that Pipkin’s does not [3–5, 7, 20].

These fractures are generally treated by either closed reduction, open reduction and internal fixation (ORIF), fragment excision, or total hip arthroplasty (THA). The surgical approach may also affect post-surgical complication rate and rehabilitation. An anterior-based surgical approach permits better access to anteriorly displaced fragments. It was previously believed that the anterior approach (i.e., Smith–Petersen) would damage the femoral head blood supply, increasing the risk of femoral head avascular necrosis (AVN), and therefore the posterior approach (i.e., Kocher–Langenbeck) was primarily used [22]. However, since the majority of the femoral head blood supply comes from the medial femoral circumflex artery; the anterior approach has no effect on the femoral head blood supply, and recent studies have utilized the Smith-Petersen (anterior) approach with successful outcomes [3, 22]. However, surgical hip dislocation (SHD), as described by Ganz et al. [23], allows for the greatest visualization and the most secure considered access to the femoral head fragments as well as being able to address the associated posterior and anterior injuries like acetabular fractures or labral tears [23–25].

Although surgical treatments and approaches have been successful for femoral head fractures, adverse outcomes such as osteoarthritis (OA), femoral head AVN, or heterotopic ossification (HO) still occur. Osteoarthritis likely occurs due to damage to the cartilaginous structures of the hip joint during the injury. Avascular necrosis, or death of bone due to interruption of bone supply, of the femoral head most often occurs due to damage to vasculature, either due to the injury itself or due to surgery. In contrast, HO most likely occurs due to trauma to soft tissues from the injury or surgery. If joint preservation options are not available, the end-stage treatment for all adverse outcomes, except HO, is typically THA [2].

Functional outcome scores are a necessary tool in determining the successful treatment of femoral head fractures as well as the patient’s long-term quality of life following an injury. Multiple different scores have been used, but the most common functional assessment tool used is the criteria developed by Thompson and Epstein [2, 3, 10, 26, 27]. Thompson–Epstein criteria are divided into roentgenographic results and clinical results. In roentgenographic classification, results were considered excellent if there was no change due to trauma, good when there were minimal changes, fair when changes were moderate, and poor when changes were severe [10]. In the more subjective clinical criteria, patients with no pain are considered to have excellent/good results, patients exhibiting moderate pain that is non-incapacitating are considered a fair result, and those with debilitating pain are considered a poor result [3, 10]. Other scores used include the Merle D’Aubigne and Postel score that is based on pain, mobility, and walking ability rated on a scale of 0–6, for a total of 18 points, and characterized as excellent, good, fair, or poor [17]. Similarly, the Harris Hip Score is based on pain, function, absence of deformity, and range of motion on a point scale from 0 to 100. The Harris Hip Score is also classified as excellent, good, fair, or poor based on numerical score. Specifically, < 70 is a poor result; 70–80 is fair, 80–90 is good, and 90–100 is excellent [28].

It remains unclear which treatment method provides the best prognosis with the fewest adverse outcomes. Because these are rare fractures, most recent publications are case reports or small case series with limited clinical follow-up. As there is no “gold standard” for classification, treatment, post-fracture rehabilitation, or outcomes for femoral head fractures, comparisons are difficult. The purpose of this systematic review and meta-analysis is to determine any changes in classification, treatment, outcomes criteria, and adverse outcomes rates for femoral head fractures since the last systematic review by Giannoudis et al. [2] in 2009 and the update in 2024 [27], and see if there have been any improvements.

## Methods

A PubMed search for publications published between January 2009 to January 2025 was conducted, as performed by Giannoudis et al. [2] Briefly, the following search terms were used: “femoral head”, “fracture”, “dislocation”, ‘‘pipkin fracture’’, “brumback fracture”, “pipkin”, and the appropriate MeSH terms: “femur head”, “fractures, bone”, “hip dislocation”. The search was limited to publications in English and studies involving humans. Once articles were selected, they were subjected to the following additional inclusion criteria (Supplement) [29], as described elsewhere [2]. An additional search of references within identified articles was also conducted. Many publications were eliminated by reading the title and/or the abstract as they did not meet the additional inclusion criteria [2]. (See outline of inclusion criteria in Table 1). Review of articles were done by authors, SM and CB, who are both orthopaedic surgery residents. The senior author (BC) adjudicated final disagreements.

**Table 1:** PICOS criteria for the study. Study quality assessment utilized the MINORS Criteria and Cochrane Risk of Bias tool for non-randomized and randomized studies/trials, respectively

Parameter	Inclusion criteria	Exclusion criteria
Patients	Non-pathological femoral head fractures associated or not with hip dislocation, regardless of treatment At least 24 months follow up At least 5 patients at final follow up in study or in femoral head fracture subgroup	Non femoral head fractures or pre-disposing pathology was described Less than 24 months follow up Less than 5 patients at final follow up
Intervention	At least one of the topics of interest was described: fracture classification (Pipkin etc.), type of treatment approach (anterior, posterior etc.)	None of the topics were described
Comparator	Either operative or non-operative treatment was described	Treatment was not described
Outcome	At least one of the following topics were described: outcome evaluation by a functional scale (Thompson and Epstein criteria); major complications (heterotopic ossification, avascular necrosis of the femoral head, or osteoarthritis)	None of the topics were described
Study Design	Publications published between January 2009 to June 2021	Case reports Systematic reviews

Some of the articles from which data was extracted consisted of patients with primarily hip joint injuries or injuries to the proximal femur. These articles were included only if femoral head fracture data was clearly separated and could easily be extracted, such as classification of fractures, treatment type and surgical approach used, and/or adverse outcomes.

Once publications met inclusion criteria, data from each publication was extracted. The age, sex, and etiology of femoral head fracture dislocation for aggregate patients were first extracted. The average age and frequency for each sex from all publications were recorded. Next, fracture classification (e.g., Pipkin), treatment type and surgical approach, adverse outcome, and functional outcome score (e.g., Thompson and Epstein) were recorded. Nonoperative and surgical treatment types, as well as surgical approach, from all publications were recorded. Adverse outcomes are defined to include HO, AVN of the femoral head, and OA. MINORS Criteria and Cochrane Risk of Bias tool were used to assess study quality for non-randomized studies and randomized studies/trials respectively [30, 31].

Meta-analyses were considered for the pooled proportion of those with femoral head fractures experiencing an adverse outcome. To determine the pooled proportion, the variances of the raw proportions from each publication reviewed were stabilized using a Freeman-Tukey-type arcsine square root transformation and the pooled proportions were calculated as the back-transform of the weighted mean of the transformed proportions, using fixed or random effects models as appropriate. Heterogeneity between studies was tested using the Q test. Random effects models were chosen if the Q test was significant; otherwise, fixed effects models were applied. Confidence intervals for adverse outcomes from individual studies are also reported.

## Results

PubMed yielded 341 English-language human study publications. Of the 341 publications, 7 met the inclusion criteria [12–15, 24, 32, 33]. After references for those articles were reviewed, 15 more eligible articles were found [21, 33–46]. In total, 22 articles were included for systematic review and meta-analysis (Table 2).

**Table 2:** Bias assessments for included articles using MINORS or Cochrane Risk of Bias. Total number of patients considered for each study are included. RCT indicates randomized controlled trial, HO indicates heterotopic ossification, AVN indicates avascular necrosis, OA indicates osteoarthritis

Assessment Criteria	Article (Year)	Type of Study	Bias Assessment	Surgically Treated	Non-surgically Treated	Classification & Type	Approach	HO	AVN	OA
	Chen et al. [14]	RCT	Low	8	8	Pipkin I	Kocher Langenback	5	2	0
*Cochrane*										
Risk of Bias	Lin et al. [35]	RCT	Some concerns regarding selection of reported result	36	0	Pipkin I	Smith Petersen	5	7	0
	Chen, Zhai et al. [13]	RCT	Low	12	12	Pipkin II	Smith Petersen	6	2	0
MINORS	Masse et al. [31]	Case series	11	13	0	Pipkin	Gibson & Kocher Langenback	2	1	0
						I, II, III, IV				
	Kokubo et al. [32]	Case series	12	10	2	Pipkin	Kocher Langenback, Smith Petersen, Watson Jones	2	0	1
						I, II, III, IV				
	Tonetti et al. [12]	Case series	12	78	32	Pipkin	Kocher Langenback, Smith Petersen, medial	0	8	0
						I, II, III, IV				
						& “Not known”				
	Park et al. [34]	Case series	12	55	4	Yoon	Kocher Langenback, Smith Petersen	7	3	13
						I, II, III, IV				
	Wang et al. [36]	Case series	13	21	0	Pipkin	Kocher Langenback			
						IV				
	Chiron et al. [21]	Case series	12	37	18	Chiron	Kocher Langenback	0	6	24
						1; A, B, C				
						2; A, B, C				
						3; A, B, C				
						4: A, B, C				
						5; A, B, C				
	Pascarella et al. [15]	Case series	12	49	20	Pascarella	Kocher Langenback, Smith Petersen, Medial, Watson Jones	1	3	12
						1; A, B, C, D				
						2; A				
						3; A, B, C, D, E, F				
						4: A, B, C				
	Mostafa et al. [33]	Case series	13	23	0	Pipkin I, II	Kocher Langenback	6	2	0
	Wanget al. [37]	Non-randomized Prospective Clinical Trial	24	39	0	Pipkin I, II	Hueter, Kocher Langenback	6	2	6
	Gavaskar and Tummala [24]	Non-randomized Prospective Clinical Trial	14	28	0	Pipkin I, II	Ganz	5	0	3
	Peng et al. [39]	Case series	13	30	0	Pipkin I, II, III, IV	Kocher Langenback, Smith Petersen	8	1	17
	Wang et al. [38]	Case series	14	12	0	Pipkin I, II, III, IV	Ganz			
	Koerner et al. [40]	Case series	14	24	4	Pipkin I, II, III, IV	Smith Petersen	3	12	8
	Shakya et al. [44]	Case Series	20	37	8	Pipkin I, II, III, IV	Kocher Langenback, Smith Petersen	4	6	16
	Hosny et al [46]	Case Series	11	18	0	Pipkin I, II	Hardinge	0	1	0
	Wang et al. [45]	Case Series	12	12	0	Pipkin III	Kocher Langenback	1	5	1
	Yoon et al. [42]	Case Series	11	42	0	Pipkin II, III, IV	Gibson	11	2	0
	Lai et al. [41]	Case Series	20	114	0	Pipkin IV	Kocher Langenback	0	18	23
	Lian et al. [43]	Case Series	12	8	0	Pipkin IV	Kocher Langenback, Smith Petersen	0	0	0

Of these 22 publications, there were three randomized controlled trials and two non-randomized prospective studies, all other studies were case series [13, 14, 24]. There was 1 study among non-randomized studies that could be deemed comparative. Among the non-randomized trials, the average MINORS score was 13.79, with scores ranging from 10 to 24. This is below the global ideal score of 15 for non-comparative studies. For the randomized trials, the overall risk of bias was low. There were some concerns of bias regarding Lin et al. [36] due to lack of variety of outcome measurements as the authors only used two measures of outcome (Thompson and Epstein and fracture reduction).

### Patient demographics

From these 22 articles, data from 810 patients with 811 femoral head fractures were reported. Of the 22 articles, 19 reported patient age and sex, while 12 studies reported the etiology of the injury. Most patients with femoral head fractures were men (50% to 100%), and the average age at time of injury ranged from 32.6 to 56 years old [12–15, 21, 32–39, 41–46]. Most fractures were due to a traffic accident (62–94.1%) [12–15, 21, 33–39, 41–46], and the follow-up period for all studies ranged from 24 to 168 months post-injury [12–15, 21, 24, 32–41, 41–46].

### Classifications

Pipkin classification was most used (Table 2). Of the 19 studies that used the Pipkin classification, Pipkin I fractures were included in 13, Pipkin II fractures were included in 13 studies and only 9 studies included Pipkin III fractures. There were 11 studies that reported Pipkin IV fractures. Chiron’s classification was used in two studies [12, 21] while Brumback’s system was applied in 1 article that reviewed 12 femoral head fractures [2, 3, 5, 20]. However, Kokubo et al. [33] used Brumback’s classification in addition to Pipkin’s. Park et al. [35] used Yoon’s modified Pipkin system, and Pascarella et al. [15] used a scheme created by the author.

### Management

The definitive treatment was identified in all 22 studies. Nine studies included participants treated non-operatively. The percentage of participants treated non-operatively ranged from 6.8 to 50% [12–14, 24, 31–33, 35–40, 44]. ORIF and fragment excision were the two most common treatment methods used with ORIF included all 22 studies. Fragment excision was reported in 13 studies and ranged from 7.1 to 50% of the patients. All 22 articles [12–15, 24, 32–40] reported the surgical approach [13–15, 32, 34, 36–38, 41–46]. The use of the Kocher–Langenbeck and Gibson approaches for SHD for the treatment of femoral head fractures is increasing as they allow reduction of all displaced femoral head fractures under direct view 47.

### Patient reported outcome measures

Reported functional outcomes varied. In 7 of the 22 articles (31.8%), the Merle D’Aubigne and Postel [17] score was used [13, 14, 24, 33, 35, 38, 46] and in six articles the Harris Hip Score was used [21, 31, 43, 44, 46]. However, Thompson and Epstein clinical criteria was most commonly used (11/22; 50%) [13–15, 32–37, 42, 45]. However, Mostafa et al. [34] combined the “excellent” and “good” into one group for Thompson and Epstein and so these were excluded.

### Adverse outcomes

**Fig. 1:**
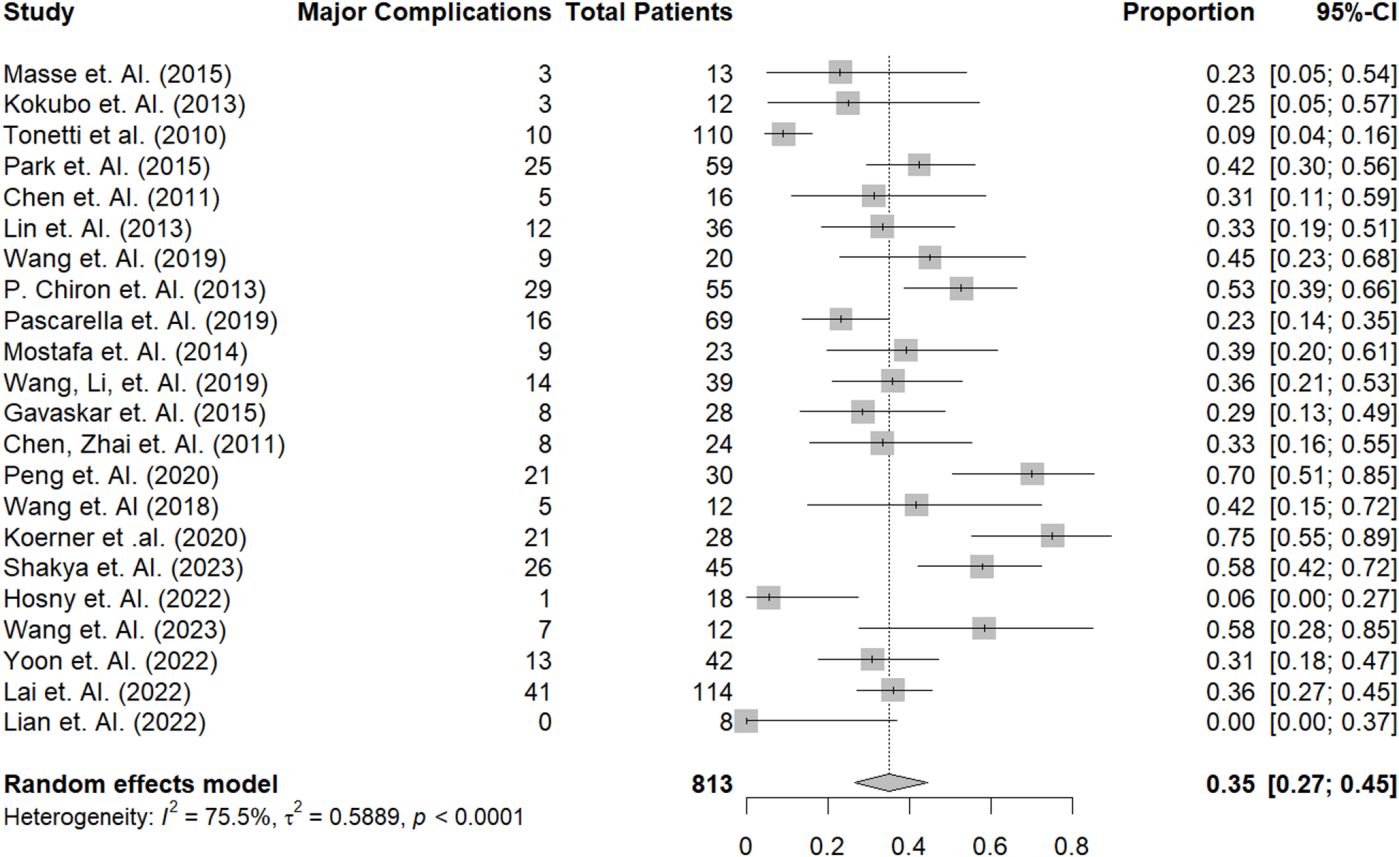
Meta-analysis results for adverse outcome rates across studies

All twenty-two articles reported adverse outcomes defined as HO, femoral head AVN, or OA. As the Q-test was significant, a random effects model was used for meta-analysis of the adverse outcome rates. The I^2^ score indicates that 75.5% of total variation across studies is due to study heterogeneity with tau squared of 0.5889 indicating the between-study variance (Fig. 1). Meta rate for adverse outcomes is 35% with 95% CI 27–45%. OA was the most common adverse outcome (up to 47.0%). AVN occurred in up to 30.7% of fractures and HO up to 27.2%. It is important to note that patients could have more than one adverse outcome.

## Discussion

Femoral head fractures are rare injuries that typically coincide with posterior hip dislocation. They most commonly result from high-energy trauma and are often associated with poor functional outcomes [2, 3, 5, 8, 36]. These injuries are more common in men [2]. The Pipkin fracture classification is the most common fracture classification system used to describe femoral head fractures [2, 3, 5, 6, 8, 10, 13–19]. Treatment of femoral head fractures can be operative or nonoperative, however, operative treatment is more common as presence of a fracture increases complexity. Although treatment varies, meta-analysis indicates that 35% of femoral head fractures have adverse outcomes of HO, femoral head AVN, or OA (95% CI, 27–45%). From this study, the lack of uniform classification criteria and differing durations of patient follow-up, indicate that there remains no “gold-standard” treatment or classification of femoral head fractures.

Fracture-dislocations of the femoral head can damage the femoral head blood supply leading to AVN. Some studies [6, 13] argue that closed reduction prior to surgery can lead to further injury to soft tissues as harmful fragments remain in the articular space and true anatomic reduction is seldom achieved [6, 13]. Other studies [8, 36, 42] show that primary closed reduction with further evaluation using computed tomography (CT) or magnetic resonance imaging (MRI) and followed by fragment excision or ORIF have more favorable outcomes than nonoperative treatment alone. In this review, operative treatment was more frequently used as the severity of fracture and the number of fragments increased. Overall, ORIF was most used, but some Pipkin III fractures were treated with THA. This is correlates with the findings of Giannoudis et al. [2] and their 2024 update [27].

Underlying anatomical variants may either predispose patients to a femoral head fracture or increase the risk of adverse outcomes. The senior author (BC) believes that preoperative imaging needs to be carefully evaluated to identify any predisposing anatomical findings consistent with femoroacetabular impingement (FAI) like acetabular retroversion, decreased femoral antetorsion, and cam and pincer lesions [48]. He believes all of these anatomical variants increases the risk of femoral head fractures with the standard mechanism of injury that causes femoral head fractures—axial loading of a flexed hip with variable hip abduction or adduction. FAI with this mechanism of injury leads to early anterior hip impingement, loading the femoral head in shear, decreasing the femoral head jump distance and correlates with the high rate of associated posterior hip dislocation.

**Fig. 2:**
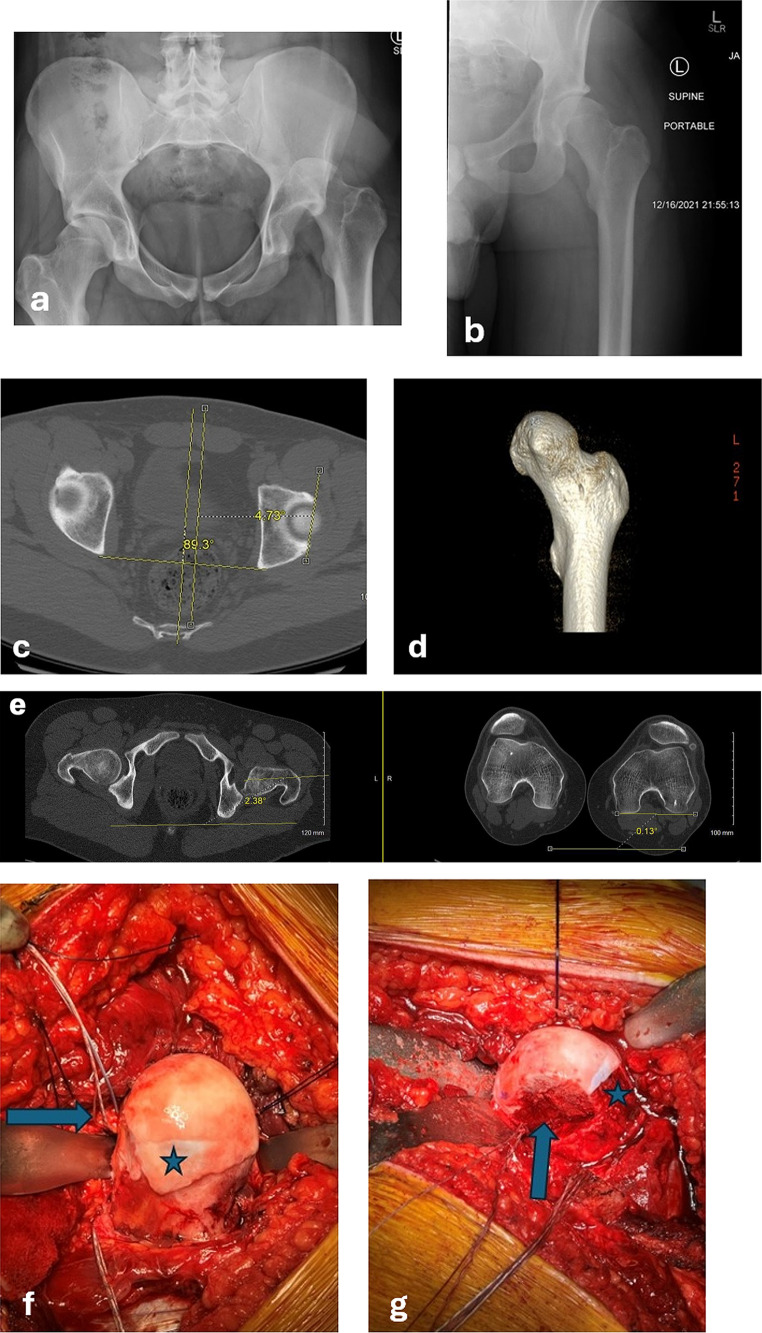

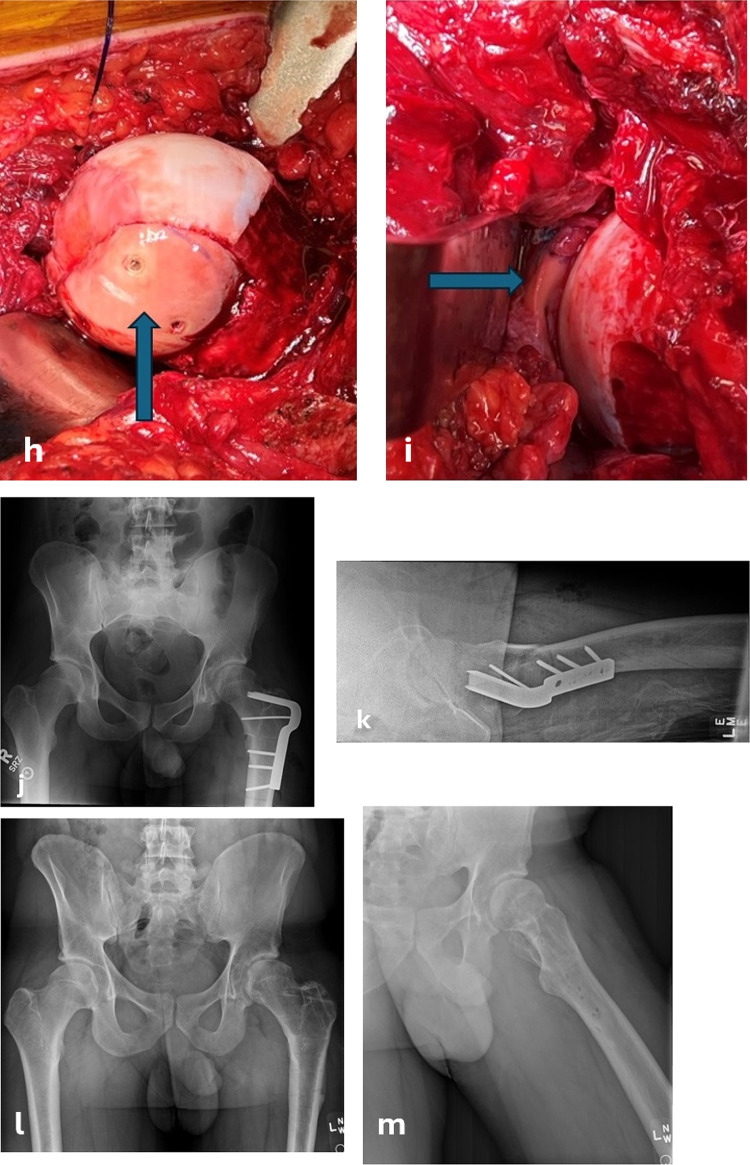
**a** Anteroposterior (AP) pelvis radiograph showing a left posterior hip fracture dislocation with an infra-foveal femoral head fracture. **b** AP hip radiograph after femoral head fragment excision. **c** CT scan after patient re-dislocated hip after fragment excision and underwent closed reduction. It shows 4.7° of cranial acetabular retroversion. **d** 3-dimensional (3D) reconstruction of the femoral head showing the infra-foveal defect after excision. **e** Axial CT cuts showing the femoral necks and distal femurs with ~ 2° of left femoral antetorsion. **f** Clinical photo showing the femoral head defect (arrow) and cam lesion (star). **g** Clinical photo showing the femoral head defect (arrow) and cam lesion (star) after SHD. **h** Clinical photo showing femoral head OCA and **i** meniscal allograft labral reconstruction. **j** Postoperative AP pelvis and lateral hip **k** radiographs after SHD, OCA, meniscal allograft reconstruction, proximal femoral antetorting osteotomy and fixation with a blade plate and screws. **l** AP pelvis and frog lateral hip **m** radiographs 17 months after the OCA surgery and 2 months after implant removal

Nonoperative treatment was most used for Pipkin I fractures. Some authors [8, 18, 19] have hypothesized that excision of a small fragment (< 1/3) of the non-weight bearing surface of the femoral head is an option for Pipkin 1 fractures. Other studies have also reported that closed reduction is a valid treatment for Pipkin I fractures [5, 9, 14]. Compared to all other Pipkin fractures, a larger percentage of all nonoperative cases were Pipkin I fractures. Of these fractures that were classified using the Thompson-Epstein Criteria, most reported having excellent or good outcomes. It is ultimately the surgeon’s discretion for treatment of these fractures based upon the severity type and prognosis. The senior author (BC) only performs fragment excision in the setting of comminuted infra-foveal fractures that are not reconstructable, otherwise ORIF is performed on all displaced infra-foveal fractures. Even in the rare setting of fragment excision, he discusses the option of doing osteochondral allograft (OCA) reconstruction of the femoral head to restore the biomechanics of the hip joint [49, 50]. Fragment excision alone in the setting of decreased femoral antetorsion and/or acetabular retroversion can lead to recurrent hip instability (Fig. 2).

It was previously [10, 19] believed that the anterior approach would damage the femoral head blood supply causing more damage. However, anatomic studies [22] showed that the majority of the blood supply to the femoral head is from the medial femoral circumflex artery and the anterior approach has no effect on the femoral head blood supply. Recent studies [12, 14, 15, 33, 35, 36] have used the Smith-Petersen approach with successful short to mid-term outcomes. An anterior approach permits better access to the femoral head as well as access to anteriorly displaced fragments when compared to the Kocher–Langenbeck approach without surgical hip dislocation (SHD). However, SHD is becoming more common because of even more improved visualization and access to both the femoral head fracture components and the associated injuries like a posterior wall acetabular fracture or labral injury [12, 14, 15, 33, 35, 36, 47]. A larger number of patients were reported utilizing surgical hip dislocation compared to Giannoudis et al. [2] The senior author’s (BC) preferred surgical approach is SHD because it provides the greatest surgical visualization and access to the femoral head and associated pathology like acetabular fractures and acetabular labral tears [25].

The high rate of adverse outcomes shown in the meta-analysis indicate improvements are needed in the treatment of femoral head fractures. For example, although many of the publications reviewed reported adverse outcomes in reference to the Pipkin fracture type, the variety of treatments makes it difficult to consider both fracture type and adverse outcome [12–15, 21, 24, 32–38, 40, 41]. In addition, other publications reporting adverse outcomes in reference to the treatment type, did not include information on the fracture type in relation to the adverse outcome. Furthermore, associated injuries like acetabular labral tears or cartilage damage are under-reported. It is rare in the senior author’s (BC) experience that there is no chondral damage associated with femoral head fractures, especially those associated with hip dislocation.

Due to the rarity of the femoral head fractures, inconsistency of evaluations, and inadequacies of classification and treatment schemes, it is difficult to provide broad recommendations and decisions regarding the classification, treatment, and outcome for these injuries. Of the current femoral head fracture studies available, few are adequate in sample size, classification criteria, treatment technique, or outcome metrics. There are very few large, multicenter studies for femoral head fractures, and those published either have inadequate patient follow-up, are too varied in fracture classification, or contain non-validated measurements. Less than two years is considered inadequate follow up as adverse outcomes can occur as long as two years after treatment [10].

Although Pipkin is the most common classification used, it is unable to classify certain types of femoral head fractures. Because of this, researchers have attempted to develop more specific systems, consequently leading to a lack of uniformity in research. As stated by Giannoudis et al. [2], future researchers must organize large, multi-center prospective studies with strict criteria in order to evaluate the classification, treatment, and outcome of femoral head fractures. However, this is highly unlikely due to the rarity of the injury. Until a large multi-center prospective observational study can be performed, either systematic review and meta-analysis, or multicenter retrospective cohort studies with pooled results may be the most effective way to determine the best treatment method and/or how to affect outcomes.

There were several limitations to this study. Because time to reduction is thought to affect risk of femoral head AVN, time elapsed between injury and reduction should be considered [2, 3]. However, similar to Giannoudis et al. [2], it was not possible to extract data regarding time to reduction because this was not uniformly documented. These results may also be dependent upon Pipkin type, associated injuries, treatment, and/or surgical approach; however, sufficient data was not available for such an analysis. Time to reduction could also be a factor for outcomes, but this was not able to be analyzed due to the variety of time to reduction reported.

## Conclusion

In 22 studies regarding the classification, treatment, and outcome of femoral head fractures, Pipkin classification was the most common classification system used. ORIF was the most common treatment method. Meta-analysis indicates that 35% of patients experienced adverse outcomes, with the most reported being OA. The high rate of adverse outcomes indicate improvements are needed in identifying the best treatment method and ways to minimize adverse outcomes. It is imperative that researchers organize large, multicenter studies. These studies must include data with uniform criteria for classification, clear conclusions about favorable treatment options, and clear standards regarding outcome.

## Data Availability

No datasets were generated or analysed during the current study.
